# A Novel Cuproptosis-Related Prognostic Gene Signature and Validation of Differential Expression in Clear Cell Renal Cell Carcinoma

**DOI:** 10.3390/genes13050851

**Published:** 2022-05-10

**Authors:** Zilong Bian, Rong Fan, Lingmin Xie

**Affiliations:** 1 Department of Big Data in Health Science, School of Public Health, Zhejiang University School of Medicine, Hangzhou 310058, China; fanrongxh@163.com; 2 Department of Surgical Oncology, Affiliated Sir Run Run Shaw Hospital, Zhejiang University School of Medicine, Hangzhou 310016, China

**Keywords:** cuproptosis, ccRCC, overall survival, progression-free survival, cell death

## Abstract

Clear cell renal cell carcinoma (ccRCC) is the most prevalent subtype of renal cell carcinoma, which is characterized by metabolic reprogramming. Cuproptosis, a novel form of cell death, is highly linked to mitochondrial metabolism and mediated by protein lipoylation. However, the clinical impacts of cuproptosis-related genes (CRGs) in ccRCC largely remain unclear. In the current study, we systematically evaluated the genetic alterations of cuproptosis-related genes in ccRCC. Our results revealed that *CDKN2A*, *DLAT*, *DLD*, *FDX1*, *GLS*, *PDHA1* and *PDHB* exhibited differential expression between ccRCC and normal tissues (|log_2_(fold change)| > 2/3 and *p* < 0.05). Utilizing an iterative sure independence screening (SIS) method, we separately constructed the prognostic signature of CRGs for predicting the overall survival (OS) and progression-free survival (PFS) in ccRCC patients. The prognostic score of CRGs yielded an area under the curve (AUC) of 0.658 and 0.682 for the prediction of 5-year OS and PFS, respectively. In the Kaplan−Meier survival analysis of OS, a higher risk score of cuproptosis-related gene signature was significantly correlated with worse overall survival (*HR* = 2.72 (2.01–3.68), log-rank *p* = 1.76 × 10^−7^). Patients with a higher risk had a significantly shorter PFS (*HR* = 2.83 (2.08–3.85), log-rank *p* = 3.66 × 10^−7^). Two independent validation datasets (GSE40435 (*N* = 101), GSE53757 (*N* = 72)) were collected for meta-analysis, suggesting that CDKN2A (log_2_(fold change) = 1.46, 95%CI: 1.75–2.35) showed significantly higher expression in ccRCC tissues while DLAT (log_2_(fold change) = −0.54, 95%CI: −0.93–−0.15) and FDX1 (log_2_(fold change) = −1.01, 95%CI: −1.61–−0.42) were lowly expressed. The expression of *CDKN2A* and *FDX1* in ccRCC was also significantly associated with immune infiltration levels and programmed cell death protein 1 (PD-1) expression (CDKN2A: *r* = 0.24, *p* = 2.14 × 10^−8^; FDX1: *r* = −0.17, *p* = 1.37 × 10^−4^). In conclusion, the cuproptosis-related gene signature could serve as a potential prognostic predictor for ccRCC patients and may offer novel insights into the cancer treatment.

## 1. Introduction

Renal cell carcinoma (RCC) is one of the most common cancer types in the urinary system affecting more than 430,000 individuals in 2020 worldwide [1]. Clear cell renal cell carcinoma (ccRCC) is the most prevalent and aggressive subtype accounting for about 70% of all RCCs [2]. Clinically, approximately a third of ccRCC patients will present with metastasis at the initial diagnosis, and a quarter of patients with localized disease will have a relapsing metastasis after curative surgical resection. ccRCC in the metastatic form is always associated with high mortality [3,4]. Therefore, given the substantial incidence and mortality of ccRCC, there is an urgent need to develop more efficient prognostic models.

Copper is an indispensable trace element involved in various biological processes. Recent studies showed that the copper levels of cancer patients are significantly elevated both in serum and tumor tissues compared to healthy counterparts [5,6,7]. While dysregulation of copper homeostasis may trigger cytotoxicity, alterations in intracellular copper levels may influence the development and progression of cancer [8]. Based on this mechanism, copper ionophores (disulfiram, dithiocarbamates, elesclomol, etc.) and copper chelators (trientine, tetrathiomolybdate, etc.) have been applied in anticancer treatment [9,10,11,12]. Recently, attention has been brought to a novel cell death pathway termed cuproptosis, and it has been proven that copper binds directly to the lipoylated components of the tricarboxylic acid (TCA) cycle, leading to toxic protein stress and, ultimately, cell death [13]. ccRCC is generally accompanied by a reprogramming of the tricarboxylic acid (TCA) cycle, downregulating the energy production through a TCA cycle and enabling tumor cells to survive in conditions of nutrient depletion and hypoxia and escape from the immune system [14,15,16]. Several genes involved in copper-induced cell death were identified, which may offer novel strategies to predict the prognosis of ccRCC patients.

In the present study, we intended to comprehensively investigate the molecular alterations and clinical relevance of cuproptosis-related genes (CRGs) in ccRCC. Our analysis highlights the importance of CRGs in ccRCC development and lays a foundation for the therapeutic application of cuproptosis regulators in ccRCC.

## 2. Materials and Methods

### 2.1. Multiomics Data Source and Preprocessing

We obtained data of cuproptosis-related genes and clinical information of ccRCC patients from the The Cancer Genome Atlas (TCGA) database (https://portal.gdc.cancer.gov//, accessed on 15 March 2022). A total of 524 ccRCC patients and 72 adjunct nontumor samples were involved in the present study. Gene expression was measured with STAR as raw read counts, which subsequently transformed into transcripts per million (TPM). All gene features (e.g., chromosome positions, gene types, Ensembl IDs and official symbols) were annotated by the GENCODE project (v22) [17]. Clinical covariates, including the overall survival outcome, age, gender, tumor stage and histological grade, were attained from the previous related resource [18]. Only ccRCC patients with survival information were included in this study.

Somatic mutation data of ccRCC from whole exome/genome sequencing (WXS/WGS) were downloaded from the GDC TCGA-ccRCC project on the UCSC Xena server [19]. The MuTect2 algorithm, which assigns higher levels of confidence to somatic variants [20], was employed to identify additional germline mutations. Oncoplot was drawn according to the descending order of mutations using the R package “maftools” [21].

Digital focal-level copy number variation (CNV) values were calculated from tumor aliquots using a “masked copy number fragment” file by GISTIC2 [22] at the item level and then cut by a noise threshold of 0.3. A Cleveland dot plot was drawn to visualize the frequency of CNV by the R package “ggpubr”.

### 2.2. Differential Expression Analysis and Validation

We investigated the differential expression levels of CRGs between tumor and normal samples. Under the criterion of |log_2_(fold change) | > 2/3 and *p* < 0.05, we considered it as statistically significant.

For validation, we collected 2 datasets (GSE40435 [23] and GSE53757 [24]) of 173 ccRCC samples from Gene Expression Omnibus (GEO). These data were generated using the platform of Affymetrix Human Genome U133 Plus 2.0 Array (GPL570)**.** Box plots were adopted to compare the expression of CRGs in various datasets using the R package “ggplot2”.

After computing the log_2_ fold change and 95% confidence intervals, we performed a meta-analysis of the results of differential expression to improve the statistical power of our study. We used the Q test (I^2^ statistics) as the assessment of the heterogeneity between multiple datasets. If there was no obvious heterogeneity (I^2^ < 50%, *p* > 0.05), a fixed effects model was chosen. Otherwise, we selected a random effects model. A forest plot was utilized to show fold change and related 95% CI of CRGs using the R package “forestplot”.

### 2.3. Gene Network and Enrichment Analysis of CRGs

To analyze the potential interactions of these genes, we performed a gene network analysis with the GENEMANIA website [25]. Furthermore, we implemented pathway enrichment analysis of CRGs with the Metascape [26] website. Gene Ontology (GO) as well as Kyoto Encyclopedia of Genes and Genomes (KEGG) were used as references, and enrichment analysis was performed by the R package “clusterProfiler” [27]. We applied the Benjamini−Hochberg method for the multiple correction, and a false discovery rate (FDR) < 0.05 was considered to be of significance.

### 2.4. Construct Prognostic Signature of Cuproptosis-Related Genes with Penalized Regression

To quantify cuproptosis-related genes at the individual level, we developed a signature based on iterative sure independence screening (SIS) [28]. We used SIS and least absolute shrinkage and selection operator (LASSO)−penalized Cox regression to screen for CRGs associated with survival using the R package “SIS” [29].

We then calculated the risk score using the regression coefficients of the identified prognostic signature of CRGs for OS and PFS, respectively. Subsequently, we classified patients into the high-risk and low-risk groups according to the median value of risk scores. The Kaplan−Meier survival curve was plotted to compare the OS or PFS between high-risk and low-risk groups by the R package “ggsurvplot”. Utilizing the signature, we also computed the 1-year survival, 3-year survival and 5-year survival based on the nearest neighbor nstimation (NNE) method [30]. Receiver operating characteristic (ROC) curves were computed for presenting the prediction ability using the R package “survivalROC”. In addition, we investigated potential differences among subgroups stratified by age, gender and tumor stage. Enhanced regression nomograms of CRG scores and other clinical covariates of ccRCC patients were constructed by the R package “regplot”. The calibration curves of the CRGs’ scores and other clinical covariates of ccRCC patients were estimated using 1000 bootstrapping to determine bias-corrected estimates of predicted versus observed values, which was analyzed using the R package “rms”.

### 2.5. Analysis of Correlation with Immune Infiltration

A Tumor Immune Estimation Resource (TIMER; cistrome.shinyapps.io/timer) [31] was used to investigate the relationship between the expression of CRGs and the abundance of six immune cells (CD4^+^ T cells, CD8^+^ T cells, B cells, neutrophils, dendritic cells and macrophages).

We also examined three important immune checkpoints (ICKs), including PD-1, PD-L1 and TIM-3 since the expression level of immune checkpoint-related genes is related to the treatment response of immune checkpoint inhibitors [32].

The Pearson correlation analysis was used to examine the association between TME or ICKs and CRGs.

### 2.6. Statistical Analysis

First, we performed a descriptive statistical analysis of ccRCC patients in TCGA. Continuous variables were described as mean ± standard deviation, and categorical variables were described by frequency and proportion. A Kruskal−Wallis rank sum test [33] was applied to examine the difference of CRG expression in various classifications of pathologic stage and histological grade of ccRCC patients.

We utilized the Benjamini−Hochberg method for multiple correction, and a FDR < 0.05 was the standard of statistical significance [34]. All statistical analyses were performed using R version 4.1.1 (The R Foundation). *p* values were two-sided, and we considered a level of *p* value less than 0.05 to be statistically significant.

## 3. Results

### 3.1. Differential Expression and Genetic Alterations of Cuproptosis-Related Genes in ccRCC

**Figure 1:**
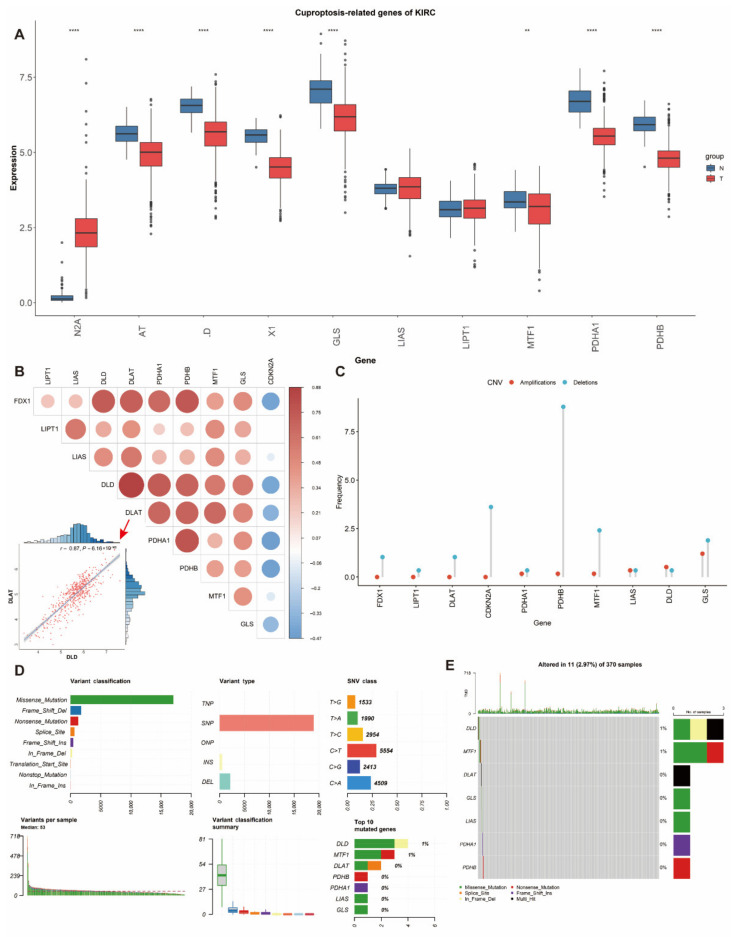
Expression and genetic alteration of CRGs in ccRCC: (**A**) the expression of 10 CRGs in ccRCC and normal tissues (tumor in red and normal in blue). The upper and lower ends of the boxes represent the interquartile range of values. The lines in the boxes represent the median value; (**B**) correlations between the expression of cuproptosis regulators; (**C**–**E**) the CNV and mutation frequency and classification of 10 CRGs in ccRCC. * *p* < 0.01, *** *p* < 0.001; CRG: cuproptosis-related gene, ccRCC: clear cell renal cell carcinoma, SNP: single nucleotide polymorphism, INS: insertion and DEL: deletion.

We curated a catalog of 10 genes (*CDKN2A*, *FDX1*, *DLD*, *DLAT*, *LIAS*, *GLS*, *LIPT1*, *MTF1*, *PDHA1* and *PDHB*) that function closely with cuproptosis [13]. In comparison of differentially expressed genes between tumor and normal tissues in ccRCC patients from TCGA, only *CDKN2A* (log_2_(fold change) = 2.12, *p* = 1.50 × 10^−155^) showed significantly higher expression while *DLAT* (log_2_(fold change) = −0.73, *p* = 5.12 × 10^−26^), *DLD* (log_2_ (fold change) = −0.97, *p* = 5.99 × 10^−47^), *FDX1* (log_2_ (fold change) = −1.07, *p* = 9.21 × 10^−54^), *GLS*(log_2_ (fold change) = −0.94, *p* = 2.85 × 10^−22^), *PDHA1*(log_2_ (fold change) = −1.14, *p* = 7.17 × 10^−32^) and *PDHB*(log_2_ (fold change) = −1.12, *p* = 2.59 × 10^−46^) showed lower expression in ccRCC tissues than normal tissues (Figure 1A, Table S1). In addition, we investigated the correlation between the expression of different genes, which revealed strong associations (Figure 1B). For instance, *DLD* was highly and positively correlated with *DLAT* (*r* = 0.87, *p* = 6.16 × 10^−163^) (Figure 1B).

The prevalence of cuproptosis-related alterations among ccRCC samples was first determined with a focus on somatic mutations and CNVs (Figure 1C–E). The CNV alterations were not universally prevalent among these genes (Figure 1C). The primary genes with CNV deletions were *PDHB* and *CDKN2A*. In contrast, *GLS* had the highest CNV amplifications. It is also interesting to notice the rare changes of *DLD* in mutation and CNV frequency. The somatic mutation status of 11 out of 370 (2.97%) ccRCC samples is shown (Figure 1D,E). According to the classification of mutations, we noticed that missense mutation was the most frequent (Figure 1D). SNP was the most prevalent variant type, and C > T (5554) ranked at the top in the single nucleotide variant (SNV) classes. We also found that *DLD* (1%) and *MTF1* (1%) showed higher mutation frequencies than others (Figure 1E).

### 3.2. Functional Enrichment and Protein–Protein Interaction Analysis of CRGs

**Figure 2:**
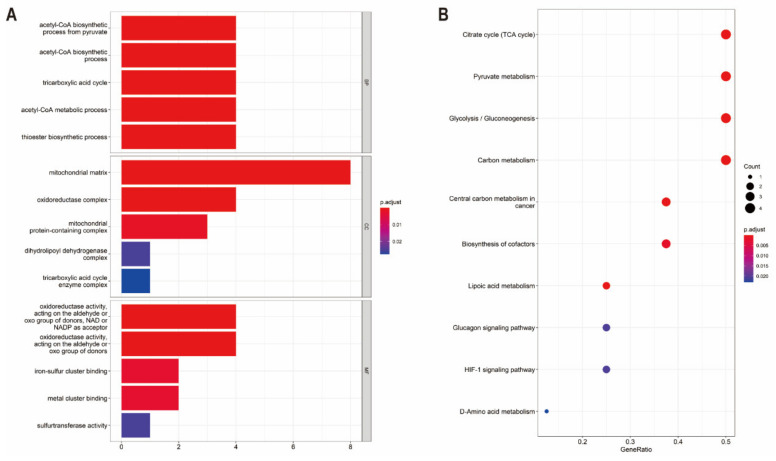
Pathway enrichment analysis of CRGs in ccRCC patients of TCGA: (**A**) the enriched item in the gene ontology analysis; (**B**) the enriched item in the Kyoto Encyclopedia of Genes and Genomes analysis. The size of circles represents the number of enriched genes. BP: biological process, CC: cellular component, MF: molecular function and CRG: cuproptosis-related gene.

To demonstrate the biological functions of CRGs, relevant pathways were analyzed by GO and KEGG databases. The biological processes of the 10 CRGs mainly involved in the GO analysis were the acetyl-CoA biosynthetic process from pyruvate, acetyl-CoA biosynthetic process, tricarboxylic acid cycle, acetyl-CoA metabolic process, thioester biosynthetic process, mitochondrial matrix, oxidoreductase complex, mitochondrial protein-containing complex, dihydrolipoyl dehydrogenase complex, tricarboxylic acid cycle enzyme complex, oxidoreductase activity, iron−sulfur cluster binding and metal cluster binding (Figure 2A). Additionally, in the KEGG pathway enrichment analysis, the 10 CRGs were largely related to the TCA cycle, pyruvate metabolism, glycolysis/gluconeogenesis, carbon metabolism, lipoic acid metabolism, central carbon metabolism in cancer, biosynthesis of cofactors, glucagon signaling pathway, HIF-1 signaling pathway and D-Amino acid metabolism (Figure 2B). A Protein–Protein Interaction (PPI) analysis was performed to explore the interactions of CRGs, showing that *DLD*, *PDHB*, *DLAT* and *PDHA1* were hub genes (Figure S1).

### 3.3. Construction of the Prognostic Signature of Cuproptosis-Related Genes in ccRCC

We further evaluated the association between the expression of CRGs and the prognosis in ccRCC. We found that all these genes except *GLS* were highly correlated with the overall survival (OS) in the univariate Cox proportional hazard regression model after the adjustment of age, gender, race and pathologic stage (Table S2). The hazards ratios of *FDX1* (*HR* = 0.54 (0.41–0.71), *p* = 1.56 × 10^−5^), *DLD* (*HR* = 0.74 (0.60–0.91), *p* = 4.10 × 10^−3^), *DLAT* (*HR* = 0.66 (0.54–0.82), *p* = 1.68 × 10^−4^), *PDHB* (*HR* = 0.70 (0.51–0.96), *p* = 2.91 × 10^−2^), *MTF1* (*HR* = 0.76 (0.61–0.94), *p* = 1.01 × 10^−2^) and *CDKN2A* (*HR* = 1.21 (1.01–1.45), *p* = 3.52 × 10^−2^) remained statistically significant (Table S2). *CDKN2A* exhibited oncogenic features, the overexpression of which was associated with worse survival in ccRCC patients (*HR* = 1.21 (1.01–1.45), *p* = 3.52 × 10^−2^, Table S2). On the contrary, high expression of the other nine genes (*FDX1*, *LIPT1*, *LIAS*, *DLD*, *DLAT*, *PDHA1*, *PDHB*, *MTF1* and *GLS*) was remarkably related to better survival in ccRCC, showing features of tumor suppressors (Table S2).

**Figure 3:**
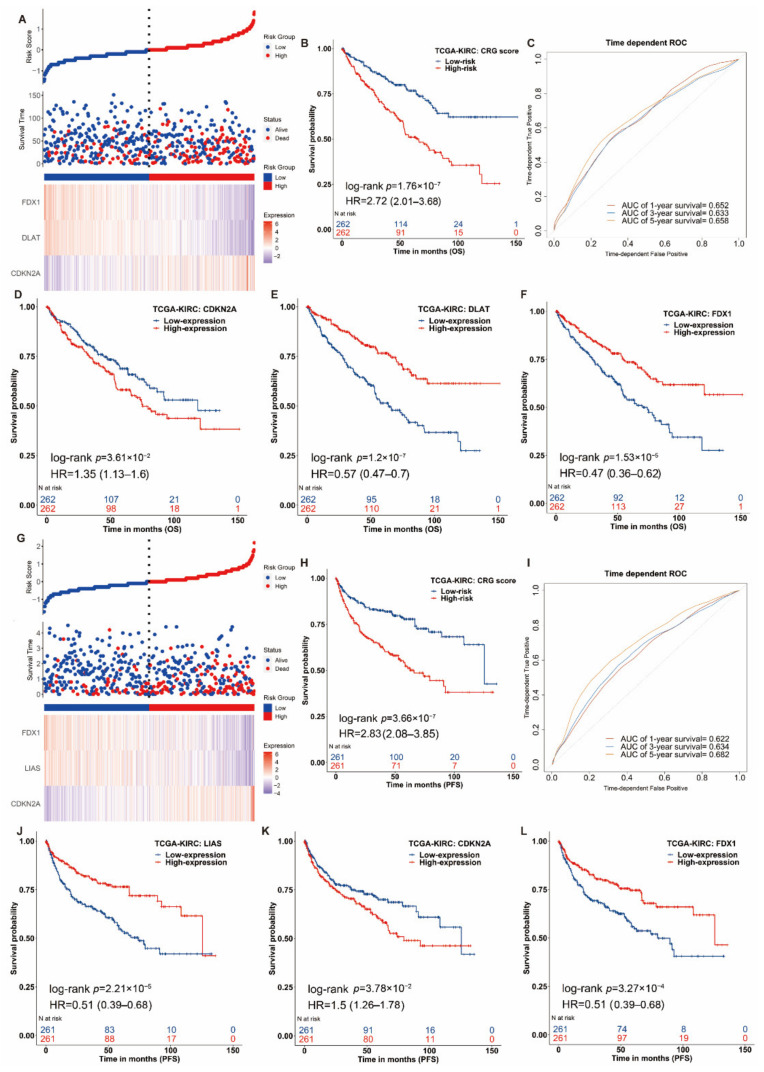
Clinical relevance of CRGs in the ccRCC patients of TCGA. For OS outcome, (**A**) distribution of risk score, survival status and the expression of prognostic CRGs, (**B**) Kaplan−Meier plot of the CRG signature and overall survival, (**C**) ROCs for one-year, three-year and five-year survival prediction. Kaplan−Meier plot for the expression of (**D**) *CDKN2A* (**E**) *DLAT* and (**F**) *FDX1* and overall survival. For PFS outcome, (**G**) distribution of risk score, status and the expression of prognostic CRGs, (**H**) Kaplan−Meier plot of the CRG signature and progression-free survival. (**I**) ROCs for one-year, three-year and five-year progression-free survival prediction. Kaplan−Meier plots of the expression of (**J**) *LIAS*, (**K**) *CDKN2A* and (**L**) *FDX1* and progression-free survival. The hazard ratios (HRs) are evaluated by Cox proportional hazard models. OS: overall survival, PFS: progression-free survival and ROC: receiver operating characteristic curve.

Then, we separately constructed the prognostic signature of CRGs for OS and PFS in ccRCC utilizing SIS. For OS outcomes of ccRCC patients, three genes were selected to construct the prognostic score using their regression coefficients: Score_os_ = −0.44 × FDX1−0.28 × DLAT + 0.23 × CDKN2A. A higher risk score of CRGs’ signature was significantly associated with poor OS (*HR* = 2.72(2.01–3.68), log-rank *p* = 1.76 × 10^−7^, Figure 3A,B). We also applied a weighted risk score incorporating all related genes to estimate 1-, 3- and 5-year OS (Figure 3C,F). The prediction accuracy evaluated by AUCs was reported to be 0.652, 0.633 and 0.658 in the 1-year, 3-year and 5-year ROC curves, respectively. For PFS, we built a risk score of cuproptosis-related gene signature using the following formula: risk score = $\overset{n}{\underset{i=1}{\sum }}Coef\;\left(Gene\right)\times Expr\left(Gene\right)$ where Coef (Gene) was the coefficient of genes (i.e., *FDX1*, *DLAT and CDKN2A*) correlated with PFS, and Expr (Gene) was the expression signature of corresponding genes. The same analyses were conducted for the PFS outcome. Patients with a higher risk score had significantly shorter PFS (*HR* = 2.83 (2.08–3.85), log-rank *p* = 3.66 × 10^−7^, Figure 3G,H), and the reported AUCs of 1-year, 3-year and 5-year ROC curves for the prediction of PFS were 0.622, 0.634 and 0.682, respectively (Figure 3G,I). In different subgroups of age, gender and pathologic stage, the CRG risk score also had a good performance (Table S3). In short, our constructed individual-level cuproptosis-related risk signature showed a significant association with survival of ccRCC.

### 3.4. Nomogram Development and Validation for ccRCC

**Figure 4:**
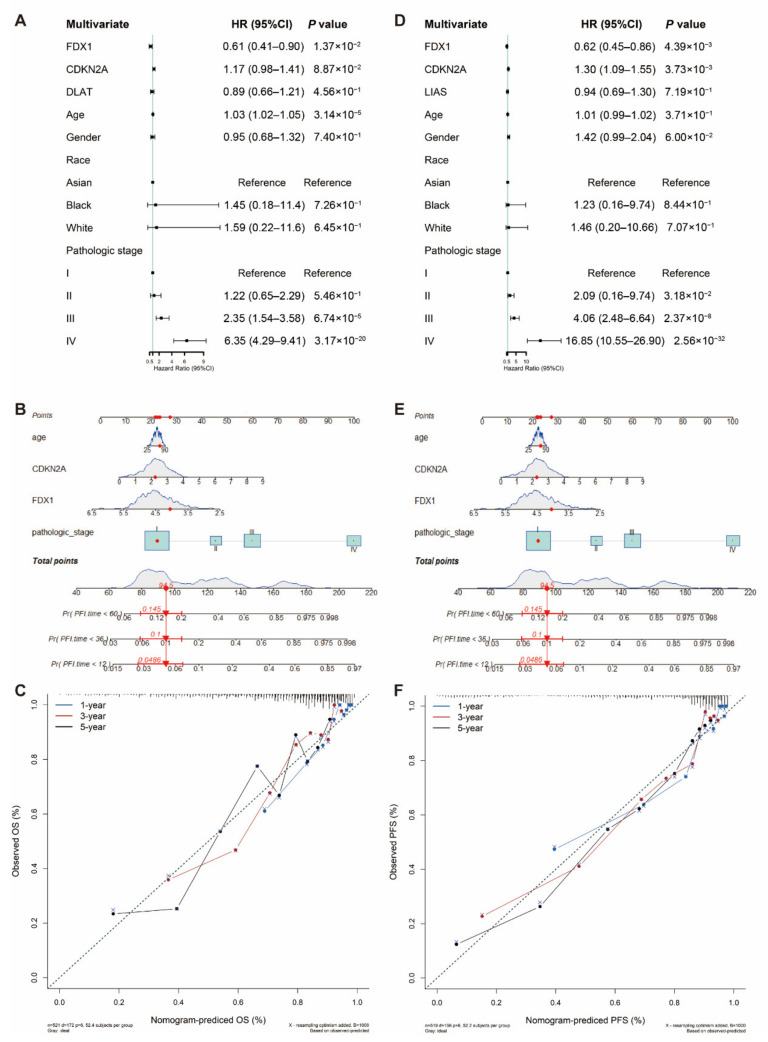
Nomogram development and validation. For (**A**) OS and (**D**) PFS, hazard ratios and *p*-value of the constituents involved in multivariate Cox regression considering clinical information and prognostic CRGs in ccRCC. Nomogram to predict the 1-year, 3-year and 5-year (**B**) OS and (**E**) PFS rate of LUAD patients. Calibration curve for the (**C**) OS and (**F**) PFS nomogram model in ccRCC. A dashed diagonal line represents the ideal nomogram. CRG: cuproptosis-related gene, ccRCC: clear cell renal cell carcinoma, OS: overall survival and PFS: progression-free survival.

To facilitate the clinical application of the prediction model, we integrated clinical information and gene features of patients from TCGA and performed the multivariable Cox regression model to develop the nomogram. Discrimination and calibration methods were applied in both OS and PFS outcomes (Figure 4). The c-index was calculated to be 0.77 for OS and 0.824 for PFS, reflecting a relatively excellent predictive performance of the nomogram. Meanwhile, calibration plots demonstrated favorable concordance between the predicted OS or PFS and the observed OS or PFS at 1, 3 and 5 years of survival (Figure 4C,F).

### 3.5. Validation of Differential Expression of CDKN2A, DLAT, FDX1 and LIAS in ccRCC

**Figure 5:**
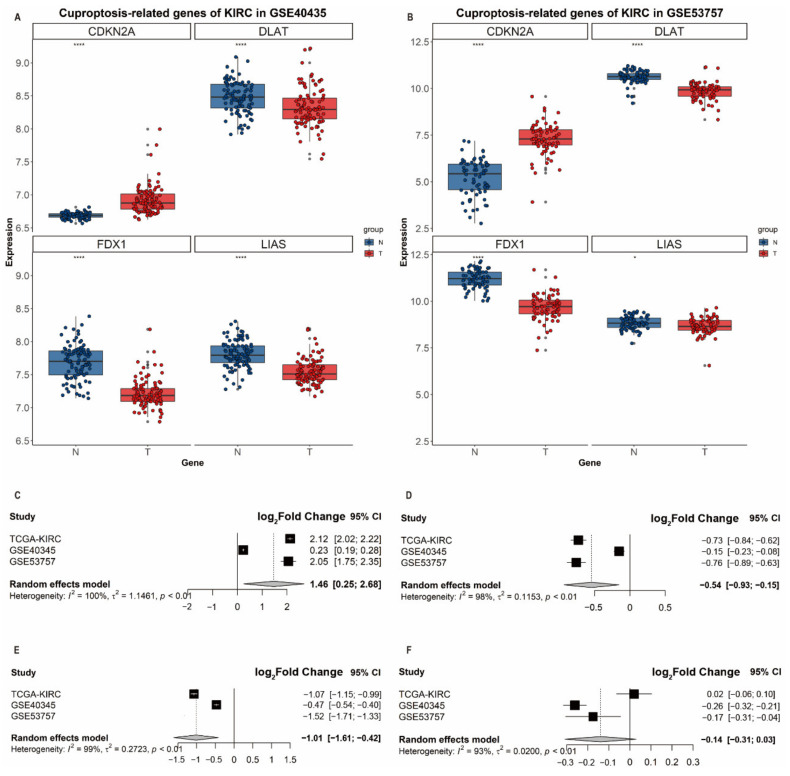
Differential expression analysis and validation in three datasets. Boxplots of the expression of *CDKN2A*, *DLAT*, *FDX1* and *LIAS* in (**A**) GSE40435 and (**B**) GSE53757. Forest plots of the meta-analysis of the differential expression of (**C**) *CDKN2A*, (**D**) *DLAT*, (**E**) *FDX1* and (**F**) *LIAS* in GSE40435, GSE53757 and TCGA.

To validate the associations of differential expression levels of identified genes with ccRCC, we collected two independent validation GEO datasets (i.e., GSE40435 and GSE53757) and performed a meta-analysis to derive the summary effect estimates. GSE40435 enrolled 101 adjacent nontumor tissues and 101 ccRCC tissues while GSE53757 included 72 normal and ccRCC tissues. In GEO datasets, *CDKN2A* showed a significantly higher expression in ccRCC tissues (GSE40435: log_2_(fold change) = 0.23, *p* = 3.76 × 10^−19^; GSE53757: log_2_(fold change) = 2.05, *p* = 5.02 × 10^−27^, Supplementary Table S1) while *DLAT*, *FDX1* and *LIAS* were significantly downregulated in their expression levels in ccRCC tissues (Figure 5A,B). Due to the presence of heterogeneity between these three datasets, we, therefore, adopted the random effects model for the meta-analysis; we found that the expression levels of *CDKN2A* (log_2_(fold change) = 1.46, 95%CI: 1.75–2.35), *DLAT* (log_2_(fold change) = −0.54, 95%CI: −0.9–−0.15) and *FDX1* (log_2_(fold change) = −1.01, 95%CI: −1.61–−0.42) (Figure 5C–F) were significantly differential between ccRCC and normal tissues, which revealed the role of *FDX1* and *DLAT* as tumor suppressor genes and the role of *CDKN2A* as a cancer promotor gene.

### 3.6. Correlation between Expression of CRGs and Immune Infiltration Levels in ccRCC

**Figure 6:**
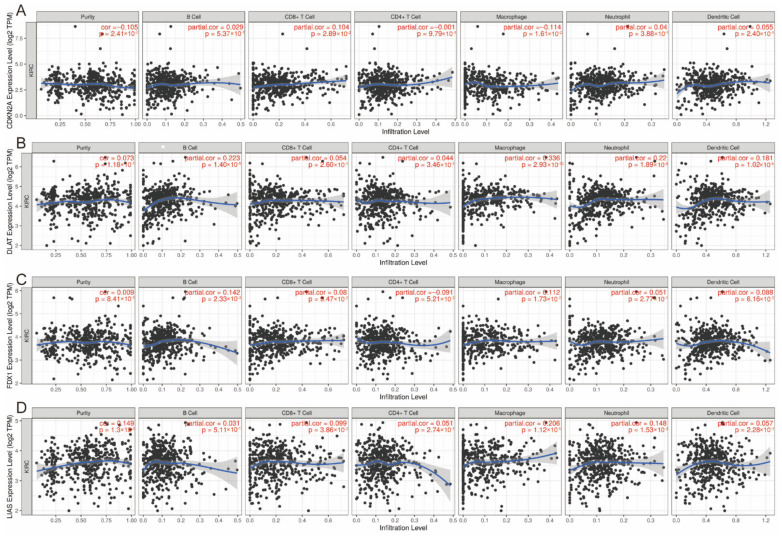
Correlation between (**A**) *CDKN2A*, (**B**) *DLAT*, (**C**) *FDX1* and (**D**) *LIAS* expression and immune infiltration in ccRCC in the TIMER database. ccRCC: clear cell renal cell carcinoma.

It is uncertain whether CRGs would influence immune cell recruitment in the tumor microenvironment and, therefore, affect the prognosis of ccRCC. Thus, we performed an analysis to examine the relationships between *CDKN2A*, *DLAT*, *FDX1* and *LIAS* and immune infiltration in ccRCC. The expression level of *CDKN2A* was positively associated with the immune infiltration level of CD8^+^ T cells (*p* = 2.89 × 10^−2^) and negatively correlated with macrophages (*p* = 2.89 × 10^−2^) (Figure 6A). The *DLAT* expression level was positively correlated with the immune infiltration level of B cells (*p* = 1.40 × 10^−6^), macrophages (*p* = 2.93E × 10^−13^), neutrophils (*p* = 1.89 × 10^−6^) and dendritic cells (*p* = 1.02 × 10^−4^) (Figure 6B). The *FDX1* expression level was positively associated with the abundance of B cells (*p* = 2.33 × 10^−3^) and macrophages (*p* = 1.73 × 10^−2^) (Figure 6C). Figure 6D presents the positive association between the *LIAS* expression and the abundance of CD8^+^ T cells (*p* = 3.86 × 10^−2^), macrophages (*p* = 1.12 × 10^−5^) and neutrophils (*p* = 1.15 × 10^−3^).

**Figure 7:**
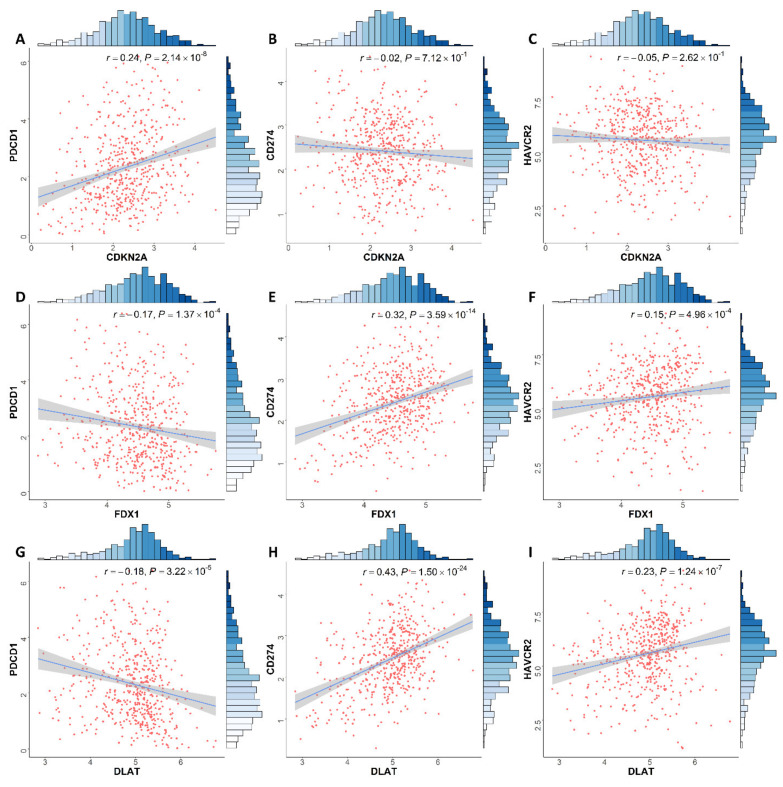
Association between (**A**–**C**) *CDKN2A*, (**D**–**F**) *FDX1* and (**G**–**I)** *DLAT* and *PDCD1*, *CD274* and *HAVCR2* expression in ccRCC patients, respectively. ccRCC: clear cell renal cell carcinoma.

Our results also showed that *CDKN2A* expression in ccRCC had a positive correlation with *PDCD1* expression levels (*r* = 0.24, *p* = 2.14 × 10^−8^). *DLAT* expression was associated with the expression level of *PDCD1* (*r* = −0.18, *p* = 3.22 × 10^−5^), *CD274* (*r* = 0.43, *p* = 1.50 × 10^−24^) and *HAVCR2* (*r* = 0.23, *p* = 1.24 × 10^−7^). *FDX1* expression has a significant association with the expression of *PDCD1* (*r* = −0.17, *p* = 1.37 × 10^−4^), *CD274* (*r* = 0.32, *p* = 3.59 × 10^−14^) and *HAVCR2* (*r* = 0.15, *p* = 4.96 × 10^−4^) from Figure 7.

### 3.7. Differential Expression of CRGs in Different Pathologic Stages and Histological Grades of ccRCC

**Figure 8:**
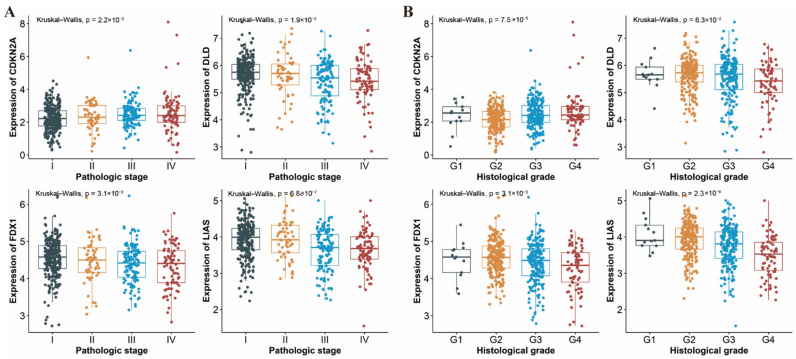
Expression of CDKN2A, DLAT, FDX1 and LIAS in a different (**A**) pathologic stage and (**B**) histological grade of ccRCC patients, respectively. ccRCC: clear cell renal cell carcinoma.

Shown in Figure 8, the expression levels of four genes (*CDKN2A*, *DLAT*, *FDX1* and *LIAS*) varied in different pathologic stages and histological grades of ccRCC, with the exception of *DLD*, which did not show any statistical difference across histological grades (*p* = 0.063, Figure 8B). Specifically, there was a downward trend of *FDX1* and *LIAS* expression and an upward trend of *CDKN2A* expression regardless of tumor stage or histological grade. These results suggested that the expression of CRGs may be correlated with disease grade and the presence of necrosis of ccRCC.

## 4. Discussion

In the present study, we explored the expression signature of 10 CRGs in ccRCC tissues and examined their relationships with OS and PFS. A novel cuproptosis-related prognostic score was constructed for the first time. In addition, functional analyses exhibited that pathways related to TCA cycle were enriched, and the CRGs were also proven to be associated with the grading and staging of ccRCC.

To the best of our knowledge, there have been no previous studies examining the correlations between CRGs and the development of ccRCC. Surprisingly, most cuproptosis-related genes were differentially expressed between tumor and normal tissues, and all these genes were significantly associated with OS and PFS, suggesting a potential role of cuproptosis in the prognosis of ccRCC and the predictive value of this score in the prediction of ccRCC survivorship.

The prognostic score constructed in this study consisted of four cuproptosis-related genes (*CDKN2A*, *DLAT*, *FDX1* and *LIAS)*. *FDX1* encodes a small iron−sulfur protein and was involved in the reduction of Cu^2+^ to Cu^1+^. In addition, *FDX1* played the role of upstream regulator in the process of protein lipoylation in the TCA cycle and key regulator of cuproptosis [13,35,36]. Dihydrolipoamide S-acetyltransferase (*DLAT*) was one of the components of the pyruvate dehydrogenase (PDH) complex. The oligomerization of DLAT was due to the integration of copper and lipoylated proteins in the TCA cycle [13]. Lipoic acid synthase (*LIAS*) encoded components of the lipoic acid pathway and synthesized a potent antioxidant termed α-Lipoic acid (LA) in mitochondria [35]. The *CDKN2A* expression functioned in the cell cycle control and was strongly correlated with the origin of a variety of tumors [36,37,38,39]. Furthermore, *CDKN2A* was shown to be absent in 76% of metastatic ccRCC samples, according to a meta-analysis [39].

Mutations leading to the overload of copper could trigger severe consequences. Nevertheless, it is feasible to manage intracellular copper levels within a certain range to selectively kill tumor cells [6]. Cuproptosis, an unconventional mechanism of cell death concerning the protein lipoylation in TCA cycle, might indicate novel insights to exploit copper toxicity to treat tumors [40]. Moreover, there have been some new findings about the pathologies of the urinary tract. Evidence has shown that there is an association between microbiota and the renal function, which is involved in the progression of several kidney diseases [41]. It has also been found that copper may contribute to the attenuation of bacterial colonization in the urethra, which may shed light on the potential therapeutic target [42]. Of interest, plant extracts contributing to the regulation and synthetization of copper-related metabolism may present as an alternative nonpharmacological intervention (NPI) strategy for cancer prevention and treatment [43,44]. For example, oregano extract could be one of the potential anticancer NPIs by invoking cell death through the mitochondrial and DNA damage pathways related to cuproptosis [44]. Additionally, plant extracts, such as the extract of the Camellia sinensis leaf, may be used to synthesize copper-related preparation and, thus, has the potential to play a part in the cancer treatment [45].

Our study has multiple advantages. Firstly, this study is the first to develop a prognostic model on the basis of CRGs. Cell death, an intense area of tumor research, has been proven fundamental to cancer origin and development [46]. Cuproptosis is a novel type of cell death that differs from any known mechanisms of cell death and is reliant on mitochondrial respiration. Such an unusual mechanism may bring up new solutions for the treatment of cancer. Moreover, compared to majority of the prognostic models which mainly focused on the overall survival, our study also took progression-free survival into consideration. PFS gain may indicate the reduction in tumor burden and symptom relief, especially for advanced cancer patients [47,48,49]. Additionally, we employed SIS to identify CRGs and adjusted covariates in both screening and testing procedures to make the results more robust.

There were several limitations in our study. First, although the validation of the expression of screened prognostic genes in TCGA and GEO demonstrated, to some extent, that three genes (*CDKN2A*, *DLAT* and *FDX1*) showed a robust prognostic effect, regrettably, no dataset with a sufficiently large sample size (*n* > 50) and clinical prognostic information was available for further validation, which is urgently warranted in future research. Second, although the prognostic score focusing on CRGs’ expression signature showed a good performance in the prediction of ccRCC survivorship, some other significant genes with predictive values were not considered in this study. Third, given that the prognostic signature was built and validated by exploiting data from public databases, further biological evidence is needed apart from the statistical evidence we offered.

In summary, this study systematically analyzed the landscape of molecular alterations and interactive genes of cuproptosis in ccRCC. Our study demonstrated that these cuproptosis-related genes may play a crucial role in ccRCC outcomes. The prognostic risk score based on the expression signature of CRGs showed a good performance for the prediction of OS and PFS of ccRCC patients and was significantly associated with immune infiltration levels and PD1 expression. Our results would also provide novel insights in developing pharmacological and nonpharmacological therapeutic strategies related to cuproptosis for cancer prevention and treatment.

## Data Availability

TCGA Data Poral: https://portal.gdc.cancer.gov/ (accessed on 15 March 2022); GEO Datasets: https://www.ncbi.nlm.nih.gov/gds/ (accessed on 18 March 2022); UCSC Xena: https://xenabrowser.net/ (accessed on 15 March 2022).

## Supplementary Materials

The following supporting information can be downloaded at: https://www.mdpi.com/article/10.3390/genes13050851/s1, Figure S1: Protein–Protein Interaction (PPI) of cuproptosis-related genes (CRGs); Table S1: Results of differential expression of cuproptosis-related genes (CRGs) in various datasets; Table S2: Association results for cuproptosis-related genes derived from univariate and multivariate Cox proportional hazards model. Table S3: Association results of the cuproptosis-related gene signature of subgroup (age, gender and pathologic stage) derived from Cox proportional hazards model.
