# Supplementary material for: A Novel Cuproptosis-Related Prognostic Gene Signature and Validation of Differential Expression in Clear Cell Renal Cell Carcinoma
Source: Genes (Basel). 2022 May 10;13(5):851. doi: 10.3390/genes13050851 (PMC9141858; doi:10.3390/genes13050851)
Supplement: Supplementary file 1 [file genes-13-00851-s001.zip › genes-1690396-supplementary.pdf]

Table S1. Results of differential expression of cuproptosis-related genes (CRGs) in various datasets

Dataset	Gene symbol	log2FoldChange	P value
TCGA-KIRC	CDKN2A	2.12	########
TCGA-KIRC	DLAT	-0.73	5.12E-26
TCGA-KIRC	DLD	-0.97	5.99E-47
TCGA-KIRC	FDX1	-1.07	9.21E-54
TCGA-KIRC	GLS	-0.94	2.85E-22
TCGA-KIRC	LIAS	0.02	6.48E-01
TCGA-KIRC	LIPT1	0.00	9.28E-01
TCGA-KIRC	MTF1	-0.29	1.38E-06
TCGA-KIRC	PDHA1	-1.14	7.17E-32
TCGA-KIRC	PDHB	-1.12	2.59E-46
GSE40345	CDKN2A	0.23	3.76E-19
GSE40345	DLAT	-0.15	7.48E-05
GSE40345	DLD	-0.51	3.02E-15
GSE40345	FDX1	-0.47	3.30E-31
GSE40345	GLS	-1.69	2.59E-48
GSE40345	LIAS	-0.26	2.09E-17
GSE40345	LIPT1	-0.02	5.06E-01
GSE40345	MTF1	-0.05	1.81E-01
GSE40345	PDHA1	-0.98	8.70E-45
GSE40345	PDHB	-1.02	1.12E-61
GSE53757	CDKN2A	2.05	5.02E-27
GSE53757	DLAT	-0.76	4.15E-21
GSE53757	DLD	-0.80	2.15E-26
GSE53757	FDX1	-1.52	3.10E-31
GSE53757	GLS	-0.61	6.31E-14
GSE53757	LIAS	-0.17	9.80E-03
GSE53757	LIPT1	-0.17	1.18E-03

Table S2. Association results for cuproptosis-related genes derived from univariate and multivariate Cox proportional hazards model.

CRGs	crude model		adjusted model	
	HR (95%CI)	P value	HR (95%CI)	P value
FDX1	0.47 (0.36-0.62)	5.64E-08	0.54 (0.41-0.71)	1.56E-05
LIPT1	0.72 (0.54-0.97)	3.19E-02	0.88 (0.64-1.22)	4.42E-01
LIAS	0.58 (0.44-0.76)	6.97E-05	0.84 (0.63-1.13)	2.57E-01
DLD	0.64 (0.53-0.78)	5.98E-06	0.74 (0.6-0.91)	4.10E-03
DLAT	0.57 (0.47-0.7)	2.18E-08	0.66 (0.54-0.82)	1.68E-04
PDHA1	0.61 (0.45-0.82)	1.33E-03	0.77 (0.56-1.06)	1.07E-01
PDHB	0.54 (0.39-0.74)	1.05E-04	0.7 (0.51-0.96)	2.91E-02
MTF1	0.69 (0.57-0.83)	1.34E-04	0.76 (0.61-0.94)	1.01E-02
GLS	0.82 (0.67-1)	5.55E-02	0.82 (0.66-1.03)	8.33E-02
CDKN2A	1.35 (1.13-1.6)	8.35E-04	1.21 (1.01-1.45)	3.52E-02

Table S3. Association results of the cuproptosis-related gene signature of subgroup (age, gender and pathologic stage) derived from Cox proportional hazards model

Subgroup	OS		PFS	
	HR (95%CI)	*P* value	HR (95%CI)	*P* value
Age				
age<65	2.96 (1.91-4.59)	1.29E-06	3.00 (2.00-4.50)	1.23E-07
age≥65	2.38 (1.56-3.63)	6.19E-05	2.78 (1.71-4.54)	4.18E-05
Gender				
Female	2.79 (1.78-4.38)	8.35E-06	3.01 (1.75-5.16)	6.67E-05
Male	2.71 (1.80-4.08)	1.81E-06	2.70 (1.85-3.94)	2.82E-07
Pathologic stage				
Early (I-II)	2.20 (1.28-3.79)	4.58E-03	3.25 (1.82-5.81)	6.73E-05
Late (III-IV)	2.18 (1.45-3.27)	1.61E-04	1.61 (1.06-2.44)	2.48E-02

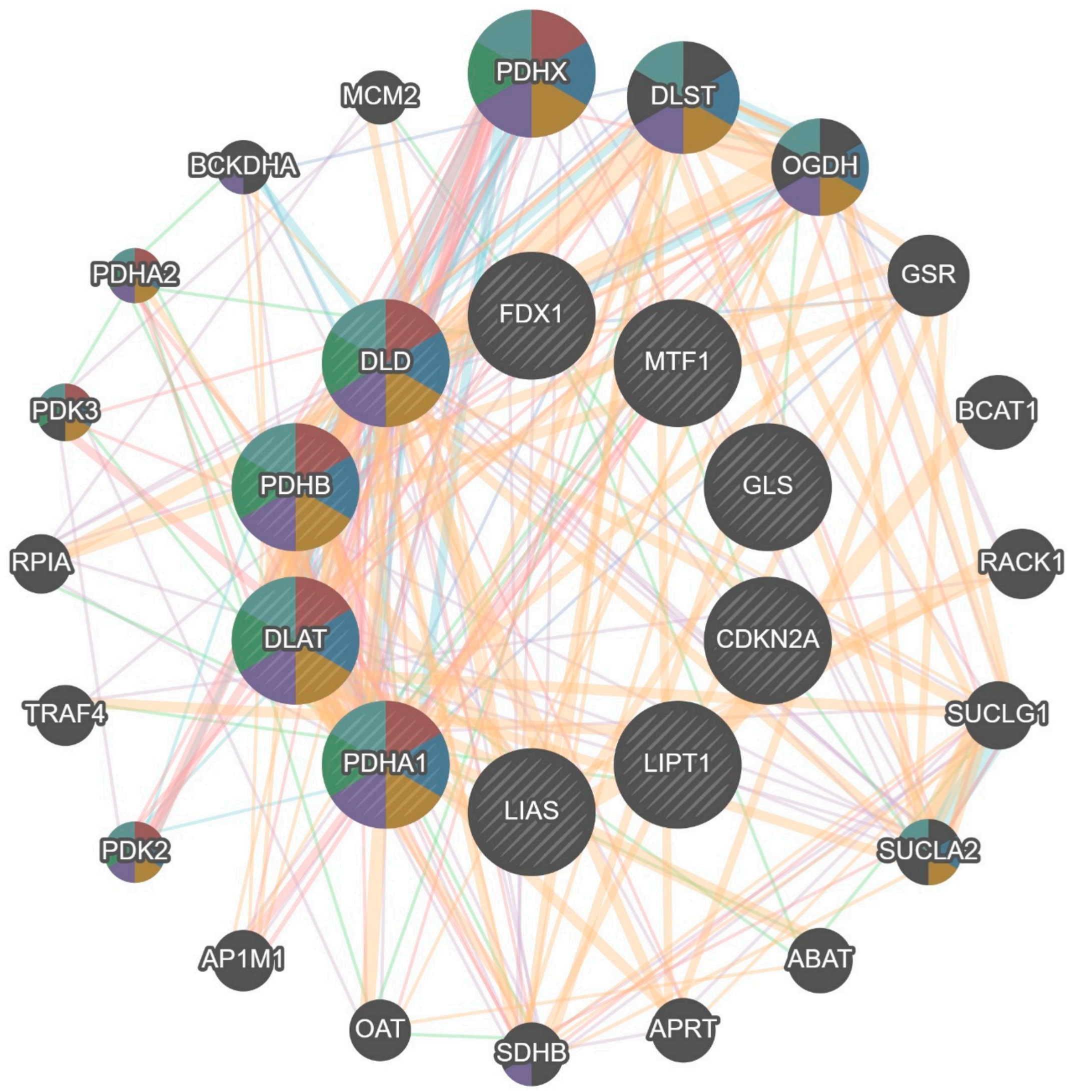

Figure S1. Protein–Protein Interaction (PPI) of cuproptosis-related genes (CRGs)
